# Inversion of Uterus in the Cow

**Published:** 1880-07

**Authors:** Quintius C. Smith

**Affiliations:** Austin, Texas


### III.—TREATMENT OF INVERSION OF THE UTERUS IN COWS, ILLUSTRATED BY TWO CASES.

Not being a veterinary surgeon, we do not presume to speak authoritatively upon this subject, but merely give the results of our experience in the two following cases, that others may profit by our experience. To be brief as possible, we note as follows: Case No. 1, large Durham cow, had been delivered—without assistance—twenty-four hours previous; found uterus inverted, hard, and much swollen. After elevating the hinder parts of the animal, the uterus being cleansed, by the combined strength of two strong men, the inversion was reduced, but to do this great and prolonged effort was necessary. The expulsive pains were so strong and frequent, that as soon as the retaining force was removed the uterus would be immediately expelled. We ordered pressure to be continued, thinking the expulsive pains would gradually cease, but they did not. After twenty-four hours of fruitless effort to retain the uterus in this manner, at the urgent request of the owner of the animal, we reluctantly consented to amputate the uterus. A strong cord was drawn tightly around the uterus, near its attachment, and each blood vessel ligated as soon as it was cut.

But the uterine tissue being brawny and resisting, it was with difficulty that the blood vessels, which were large and numerous, could be drawn out sufficiently for ligation. The uterus being removed, the "stump" diminished so rapidly in volume that many of the blood vessels required re-ligation.

The bleeding points being all secured, and the parts replaced, the animal was ordered to have a drink of salted water. The animal endured the operation well, and for a few hours promised to recover, but subsequently died, six or eight hours after the operation. Post-mortem examination proved that internal hemorrhage was the cause of death.

Many of the ligatures were loose. This case occurred in the month of August. Case No. 2 was much like No. 1, except that it occurred in cold weather. Warned by our previous failure, we determined to pursue a different plan of treatment in this case. The uterus had remained inverted all night, the weather being quite cold; the cow having been delivered eighteen hours previously. The animal was greatly exhausted, her uterus large, hard and intensely congested. After elevating the hinder parts of the animal, and cleansing the uterus, we wrapped the uterus in wet, hot blankets, which we kept heated by pouring water of as high temperature as could be borne by the hand, over them. After persevering in this treatment for about two hours, the uterus became soft and flabby, and presented a blanched and shriveled appearance. We then reduced the inversion, with comparative ease, replaced the parts in their natural position, and syringed the uterus with cold water for fifteen or twenty minutes. Our object in using the cold water injections, was to restore the natural resiliency of the uterine tissues, and thereby cause the uterus to remain in its proper position. This case gave no further trouble, and the animal promptly recovered.

QUINTIUS C. SMITH, M.D.,

March, 1880. Austin, Texas.

---

NOTE.—We suggest that in cases like No. 1 it would be well to try the effect of a tampon and full doses of chloral hydrate, combined with opium. We usually administered the following:

R.—Chloral hydrate, 1½ oz.

Tr. of opium, 1½ oz.

Warm milk, 1 qt.

Repeat every hour till pains cease. Occasionally it is necessary also to tampon and plug the vagina with cotton or tow after the reduction of the inversion retaining the tampon in position by means of a compress and strap attached to a collar around the patient's neck.—ED.

